# Heart Rate Variability in Children and Adolescents with Autism, ADHD and Co-occurring Autism and ADHD, During Passive and Active Experimental Conditions

**DOI:** 10.1007/s10803-021-05244-w

**Published:** 2021-10-30

**Authors:** Alessio Bellato, Iti Arora, Puja Kochhar, Danielle Ropar, Chris Hollis, Madeleine J. Groom

**Affiliations:** 1 Division of Psychiatry and Applied Psychology, School of Medicine, University of Nottingham, Institute of Mental Health, Innovation Park, Triumph Road, Nottingham, NG7 2TU UK; 2 Child and Adolescent Mental Health Services (CAMHS), Derbyshire Healthcare NHS Foundation Trust, Temple House, Mill Hill Lane, Derby, DE23 6SA UK; 3 School of Psychology, University of Nottingham, University Park, Nottingham, NG7 2RD UK; 4 Mental Health & Clinical Neurosciences, School of Medicine, University of Nottingham, Nottingham, UK; 5 NIHR MindTech Medtech Co-operative, Institute of Mental Health, Innovation Park, Triumph Road, Nottingham, NG7 2TU UK; 6 NIHR Nottingham Biomedical Research Centre, Institute of Mental Health, Innovation Park, Triumph Road, Nottingham, NG7 2TU UK

**Keywords:** ADHD, Autism, Autonomic nervous system, Heart rate variability, Comorbidity, Cognitive function

## Abstract

Despite overlaps in clinical symptomatology, autism and ADHD may be associated with opposite autonomic arousal profiles which might partly explain altered cognitive and global functioning. We investigated autonomic arousal in 106 children/adolescents with autism, ADHD, co-occurring autism/ADHD, and neurotypical controls. Heart rate variability was recorded during resting-state, a ‘passive’ auditory oddball task and an ‘active’ response conflict task. Autistic children showed hyper-arousal during the active task, while those with ADHD showed hypo-arousal during resting-state and the passive task. Irrespective of diagnosis, children characterised by hyper-arousal showed more severe autistic symptomatology, increased anxiety and reduced global functioning than those displaying hypo-arousal, suggesting the importance of considering individual autonomic arousal profiles for differential diagnosis of autism/ADHD and when developing personalised interventions.

**Supplementary Information:**

The online version contains supplementary material available at 10.1007/s10803-021-05244-w.

## Introduction

Autism Spectrum Disorder (ASD, from hereon, autism) and Attention Deficit/ Hyperactivity Disorder (ADHD) are neurodevelopmental conditions that typically manifest in early childhood. While ADHD is mainly characterised by inattention and hyperactivity/impulsivity, autism is defined by social communication and interaction difficulties, and restricted and repetitive behaviours, interests or activities (American Psychiatric Association, 2013). Autistic and ADHD symptomatology are associated with each other, with overlapping symptoms of inattention, reduced joint attention, and sensory processing atypicalities found in both conditions (Johnson et al., 2015; Krakowski et al., 2020; Visser et al., 2016). Shared risk factors and a high percentage of co-occurrence have also been reported (Rommelse et al., 2010). Identifying underlying factors that differentiate autism and ADHD may help to provide important individual targets for intervention in these neurodevelopmental conditions.

A domain which might differentiate ADHD from autism is arousal regulation, namely the physiological mechanisms that characterise alertness, wakefulness, and reactivity to the environment (Lacey, 1967). Synergistic interactions between the central nervous system (CNS) and peripheral nervous systems (including the autonomic nervous system; ANS) are essential for supporting higher-level cognitive and adaptive functions in humans. The two branches of the ANS, the sympathetic nervous system (SNS) and the parasympathetic nervous system (PNS), act synergistically to regulate arousal according to the demands of the environment. More specifically, the SNS is more active in situations that require fast mobilisation of energetic resources to carry out *fight-or-flight* behavioural responses (e.g., bodily reactions to a threat or danger, when activity of the parasympathetic branch is inhibited). Conversely, the PNS is active (and predominant over the sympathetic branch) when energetic resources should be preserved for a period of time, including those situations that require sustained attention or *rest-digest* behavioural responses (e.g., relaxing, healing and reducing stress levels).

Atypical functioning or regulation of one or both branches of the ANS, leading to reduced or heightened arousal, are likely to negatively impact performance on cognitive tasks (Yerkes & Dodson, 1908). For example, although easier and repetitive tasks might require less effort to be completed (at least initially), when they become less engaging and boring they may require the implementation of strategies aimed at up-regulating autonomic arousal to sustain attention. Similarly, more mentally challenging tasks are likely to elicit a state of hyper-arousal, due to their engaging and challenging nature; however, if arousal is not properly regulated to meet the demands of the task or activity, increased physiological stress and anxiety might become detrimental for performance.

Autism and ADHD have been proposed to be associated with different atypicalities in the domains of arousal and arousal regulation in specific situations or experimental contexts, and these differences can be relevant in the onset and development of these conditions (Howells et al., 2012; Klusek et al., 2013; Orekhova & Stroganova, 2014). For example, people with ADHD have been found to display signs of hypo-arousal, particularly during more monotonous and less engaging situations (Bellato et al., 2020). In these contexts, the hyperactive and impulsive behaviours that characterise ADHD might be a compensatory mechanism to upregulate arousal (Geissler et al., 2014). Conversely, during activities which are presented at an optimal pace (Wiersema et al., 2006) or are associated with external incentives and rewards (Groom et al., 2013), individuals with ADHD demonstrate better cognitive performance, possibly due to more optimal arousal regulation under these conditions (Sergeant, 2000, 2005).

Findings from research on autonomic arousal in autistic individuals are less clear, with evidence of both hyper- and hypo-arousal during resting-state (Arora et al., 2021) and during tasks requiring passive attention to sensory and social stimuli (Lydon et al., 2016). Studies on autonomic arousal measured during cognitively demanding tasks (i.e., challenging higher-level executive functions such as response-inhibition or task-switching) are less prevalent in the autism literature. However, there is emerging evidence that cognitive tasks might elicit hyper-arousal in autistic individuals: for example, increased heart rate was found before and during cognitive tasks requiring visual/auditory processing or eliciting a stress response, e.g., the Stroop task (Guy et al., 2014; Kushki et al., 2013; Porges et al., 2013). Importantly, some studies identified autistic subgroups with opposite autonomic arousal profiles (i.e., hyper- and hypo-arousal) which were associated with different profiles of adaptation and responsiveness to stimuli (Hirstein et al., 2001; Mathersul et al., 2013; Schoen et al., 2008). There is also some evidence that at least a subgroup of autistic individuals displays reduced parasympathetic vagal tone, leading to hyper-arousal, and this is linked to socialization difficulties, atypical development of language and communication skills, and internalizing and externalizing symptoms in autism (Bazelmans et al., 2019; Cai et al., 2019; Patriquin et al., 2013).

Taken together, these findings suggest that: i) less stimulating and less challenging experimental contexts might trigger hypo-arousal in individuals with ADHD, ii) at least a subgroup of autistic individuals might display a profile of hyper-arousal during more cognitively demanding tasks and iii) another subgroup of autistic individuals might be characterised by hypo-arousal. The autonomic arousal profiles of individuals with co-occurring autism and ADHD (those who meet the diagnostic criteria for both conditions) have not been investigated previously, to our knowledge.

The first aim of the present study was to investigate differences in autonomic arousal between individuals with autism, ADHD and neurotypical children, in different experimental contexts. Further, we investigated the autonomic arousal profile of individuals with co-occurring autism and ADHD, to determine whether this phenotype is associated with the same atypicalities found in those with a single diagnosis of autism or ADHD (*additive* model of comorbidity) or if it is independent from those groups, reflecting a unique profile (*interactive* model).

In a sample of children and adolescents, we recorded heart rate and calculated heart rate variability as measures of autonomic arousal. Heart rate variability (HRV) has been proposed to reflect the overall activity of the SNS and PNS but, more specifically, to be primarily influenced by parasympathetic activity and top-down control of the PNS (Thayer et al., 2012). Increased HRV has been associated with better sustained attention (Suess et al., 1994), more effective behavioural inhibition (Porges, 2007, 2009), better emotional regulation (Gentzler et al., 2009) and increased adaptive functioning (Shaffer & Ginsberg, 2017). In this study, we used non-linear measures of HRV proposed by Toichi et al (1997), the cardiac sympathetic index (CSI) and the cardiac vagal index (CVI), to index variability and activation in the SNS and the PNS respectively (see Online Resource 1, ESM_1, for more details on these measures). We also calculated average heart rate to index overall arousal levels. Higher CSI and HR indicate hyper-arousal, while higher CVI indicates reduced autonomic arousal.

We measured HRV in three different experimental contexts. First, participants took part in a short resting-state measurement, during which they were asked to relax and watch a silent film. Thereafter, they completed a passive auditory oddball task, during which simple auditory stimuli played in the background as the participants continued to watch the silent film. As there were no demands on cognition and they were asked to passively pay attention to the auditory stimuli, we describe this as a ‘passive’ task. Finally, a response conflict task was used: participants were presented with directionally congruent and incongruent visual stimuli and were required to make a button-press response on each trial. This task involved sustained attention and resolution of response conflict on incongruent trials; we therefore define this as an ‘active’ cognitive task. We expected that participants with ADHD would display indices of autonomic hypo-arousal, especially during less stimulating conditions such as at rest and during the passive task. Moreover, we expected that autistic participants would display a less homogeneous group profile at rest and during the passive task, but that they would display hyper-arousal during the active response conflict task (more specifically, reduced parasympathetic activity).

In addition, using data from the same study, we used cluster analysis to determine whether transdiagnostic sub-groups could be identified based on their autonomic profile. This approach was informed by the RDoC framework (Cuthbert & Insel, 2013), which proposes to focus on transdiagnostic domains of human functioning to better understand mental health conditions and symptoms. In our study, we focussed on the association between the domain ‘Arousal/Regulatory system’ and behavioural measures of functioning, to investigate whether modelling individual differences in autonomic arousal could contribute to a better understanding of the clinical heterogeneity within the diagnostic groups of autism and ADHD. We expected to classify our participants in at least two groups, one with a profile of hyper-arousal and one with a profile of hypo-arousal, and we aimed to compare the clusters on different behavioural and clinical measures, including ADHD and autistic symptomatology, sensory profile and global functioning.

## Methods

### Sample Characteristics, Recruitment and Ethical Approval

The present paper is based on data collected for the SAAND study, carried out at the University of Nottingham UK between September 2017 and May 2020. Ethical approval for the main study was obtained from the UK National Research Ethics Committee and the Health Research Authority. Children between 7 and 15 years of age diagnosed with or under clinical assessment for autism and/or ADHD were recruited from local support groups or were referred by paediatricians, child and adolescent psychiatrists or mental health nurses in NHS paediatric clinics and child and adolescent mental health services (CAMHS), or local special educational needs teams in both mainstream and special schools. Neurotypical participants were recruited from local schools and from a database of volunteers held by the School of Psychology, University of Nottingham UK. The study was advertised on social media platforms and information about the study was shared on an online blog published by the Association for Child and Adolescent Mental Health (ACAMH). Children under pharmacological treatment for ADHD with stimulants were required to withdraw their medication for at least 24 h before the testing session. Potential participants were excluded if they had any neurological conditions; if they were on non-stimulant medication (for example, atomoxetine, guanfacine or clonidine); if they or their parent/legal guardians were unhappy with stimulant medication being withdrawn for 24 h prior the testing session; or if they did not speak fluent English. Children were not excluded if they had a co-occurring diagnosis of mental health or behavioural conditions (including anxiety, depression, oppositional defiant or conduct disorder), or intellectual disability (i.e., IQ < 70). Children recruited as neurotypical controls were not included in the study if they were siblings of a child with a pre-existing diagnosis of autism, ADHD or any other ICD-10/DSM-5 psychiatric diagnoses.

### Clinical Assessment

Symptoms of ADHD and autism were evaluated for all participants using parent- and teacher-report Conners’ Rating Scales (CRS-3; Conners, 2008) and Social Communication Questionnaire (SCQ; Berument et al., 1999; Rutter et al., 2003), respectively. Further, the Autism Diagnostic Observation Schedule, Second Edition (ADOS-2; Lord et al., 2012) was administered by one of the authors (IA, accredited with research reliability) to all study participants referred to the clinical groups (autism or ADHD). All ADOS-2 administrations were video-recorded and those scoring near the threshold of Autism spectrum were double-rated by a Consultant Child & Adolescent Psychiatrist with research reliability accreditation for the tool and experience in Autism (PK), to confirm the presence of clinically significant features of autism. The Development and Well-Being Assessment (DAWBA; Goodman et al., 2000) and the Strengths and Difficulties Questionnaire (SDQ; Goodman, 2001) were completed online by the parents of every child (referred to both clinical and neurotypical groups). These provided a computer-generated profile of children and adolescents’ behavioural and emotional symptoms, and prosocial behaviours; and were used to guide the research classification of autism, ADHD and other ICD-10 and DSM-5 psychiatric diagnoses by PK (an experienced child and adolescent psychiatrist) (McEwen et al., 2016). The Children’s Global Assessment Scale (CGAS, Shaffer et al., 1983) was rated by one of the authors (IA), based on the DAWBA, and these scores were used to represent a measure of each child’s global functioning. The parents also completed the Child Sensory Profile 2 (Dunn, 2014) to provide a measure of their child’s sensory processing profile. The Wechsler Abbreviated Scale of Intelligence (WASI-II; Wechsler, 2011) was administered to each child to obtain a measure of their intellectual functioning. All these instruments were chosen under the supervision of a Consultant Child and Adolescent Psychiatrist (CH), who recommended their use for the high validity and reliability, and the wide use in research on autism and ADHD. A consensus diagnosis of autism and/or ADHD was confirmed by two clinicians (PK and CH) using DSM-5 criteria and combining all available information, including the DAWBA interview, parent- and teacher-reports and the child’s behavioural assessment.

### Experimental Tasks

The testing session of the main SAAND study was subdivided into two batteries of experimental tasks with (a) eye-tracking and (b) EEG. The present paper is focused on measures collected during the EEG battery only, which comprised a 3-min resting-state period, a ‘passive’ auditory oddball task and an ‘active’ response conflict task. A detailed description of the EEG setup, data collection and analyses (not presented in this paper) can be found in Bellato et al. (2021), where we presented the main results from the EEG ‘active’ task.

#### Resting-State

At the beginning of the EEG battery, a 3-min resting-state period measurement was obtained. During this time, children watched a silent movie (“Despicable Me”) and were asked to relax and sit still.

#### Passive Auditory Oddball Task

After the 3-min resting-state period, a passive auditory oddball task that lasted 25 min was presented to the participants, who continued watching the silent movie and were instructed to ignore the sounds. A series of repetitive 200-ms auditory stimuli (‘standard’; 500 Hz sinusoidal tone), alternated by less frequent stimuli (‘deviant’), was presented at an inter-stimulus interval of 700 ms. Deviant tones were either social (resembling the English vowel /e/; created with the Simplified Vowel Synthesis Interface, http://www.asel.udel.edu/speech/tutorials/synthesis/vowels.html) or non-social (450 Hz sinusoidal tone). The type of deviant (social or non-social) was manipulated due to evidence that autistic individuals might demonstrate reduced salience for social information (Chita-Tegmark, 2016). However, considering that the results of this analysis are beyond the scope of this article, we did not model the social/non-social factor in the analyses presented here. Social and non-social deviants were presented in two separate 12-min blocks of the task (block 1 and block 2), and the type of deviants in the first or second block was randomised and balanced across participants. Each block was preceded by a 30-s pre-block ‘break’ during which children continued watching the movie, but no sound was presented.

We predicted that neurotypical participants would demonstrate an initial sympathetic response to the auditory stimuli (indexed by higher CSI during the task blocks vs breaks), but that they would not exhibit parasympathetic activation (indexed by no changes in CVI between breaks and task blocks) since no demands were placed on attention or response preparation. We predicted that individuals with ADHD would demonstrate a sympathetic hypo-arousal profile and will exhibit a diminished sympathetic response to auditory stimuli (i.e., reduced CSI). We did not expect group level differences in autistic participants in this task, in line with the literature and the predicted heterogeneous autonomic arousal profiles in autistic participants when cognitive demand is low.

#### Active Response Conflict Task

**Fig. 1:**
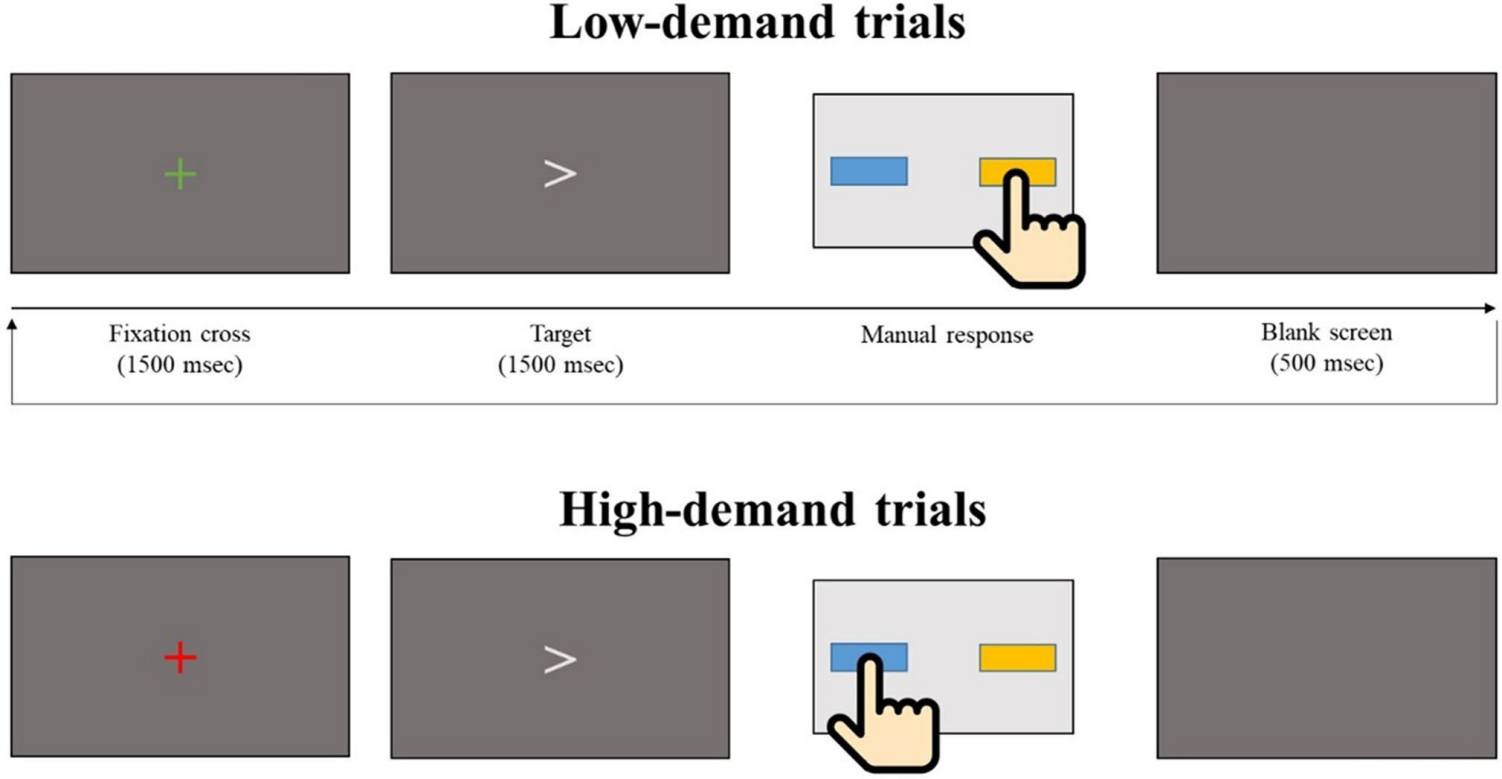
POP task diagram

After the oddball task, we presented an adapted version of the Preparing to Overcome Prepotency task (POP task; Cho et al., 2006), a response conflict task where participants were required to actively respond to task-relevant stimuli and sustain attention for about 20/25 min. Children were instructed to press the left or right button on a response box as soon as possible after the appearance of a target, a left or right arrow. In half of the trials, a cue preceding the arrow was a green fixation cross, indicating that the motor response required after target-onset should have been congruent with the arrow direction (for example, pressing the right button in response to the right arrow; ‘low-demand trials’). In the other half of trials, the cue was a red fixation cross, indicating that the behavioural response required after target presentation had to be contralateral to the direction of the target arrow (for example, pressing the left button if the red fixation cross was followed by a right arrow; ‘high-demand trials’) (see Fig. 1 for the task diagram). The presentation of cue- and target-stimuli lasted for 1500 ms each, with 500 ms of blank screen between the offset of targets and the start of the next trial. The overall task consisted of two main halves (1st half and 2nd half), which had 4 task blocks each (i.e., 8 blocks in total). Each block had 36 trials (288 trials in total), and at the end of each block there was a 50-s break followed by a 10-s visual countdown which indicated the re-start of the task. There was a short interval (30-to-60 s) between the first and the second halves of the task (i.e., between the end of the break after the 4th task block and the beginning of the 5th task block), during which children’s comfort was checked.

Considering that sustaining attention and performing mentally challenging tasks is likely to be accompanied by increased parasympathetic activation (Minzenberg et al., 2008), we expected to observe significant differences between the task blocks and the breaks in relation to CVI and CSI. Specifically, we expected that CVI would be increased during the blocks of the task compared to the breaks. In line with the main hypotheses of the study, we expected to observe reduced parasympathetic activation in autistic children compared with non-autistic children (i.e., reduced CVI during the task) while, due to the ‘active’ nature of the task, we did not expect to observe dysregulated arousal in those with ADHD.

### Procedure, Data Processing and Outcome Measures

During the EEG session, heart rate (HR) was recorded from two free electrodes placed on participants’ wrists. Raw HR signal was processed to obtain non-linear measures of HRV, the Cardiac Sympathetic Index (CSI) and the Cardiac Vagal Index (CVI). To derive these measures, heart rate signal was band-pass filtered (8–20 Hz) to minimise the impact of artifacts and high frequency noise (Fedotov, 2016), before automatic detection of cardiac beats was carried out in Brainstorm (Tadel et al., 2011). This was followed by visual correction of potentially erroneous or missing peaks, before calculating the inter-beat intervals (IBI), i.e., the time differences between successive heartbeats. Full procedure and mathematical formulae used to calculate CSI and CVI, are presented in Online Resource 1 (ESM_1).

### Analysis Plan and Statistical Tests

The effects of ADHD and autism were investigated using the binomial between-subjects factors of ADHD and Autism (0 = absent; 1 = present) reflecting the presence (or absence) of a diagnosis of ADHD or autism. In this way, we could compare children with and without ADHD (0: NT and autism-only; 1: ADHD-only and autism + ADHD), and those with or without autism (0: NT and ADHD-only; 1: autism-only and autism + ADHD), to test specific effects of each condition on the outcome measures. Moreover, we could investigate the impact of co-occurring autism and ADHD by following up significant interactions between the ADHD and Autism factors or the co-presence of significant main effects of ADHD and Autism. When following-up significant interactions between ADHD and Autism factors, we calculated p-values adjusted for multiple comparisons using the Benjamini-Hochberg (BH) method (Benjamini & Hochberg, 1995). Greenhouse–Geisser adjusted degrees of freedom (Greenhouse & Geisser, 1959) are reported for those variables for which we found violation of sphericity, which was evaluated through Mauchly’s tests.

Considering that our main interest was to compare groups of participants within each context, we initially carried out separate analyses for each experimental context (resting-state, passive oddball task, active response conflict task). In this way, we could also maximise the number of participants included in each of these analyses (some children only completed the resting-state and passive tasks, but not the active task). However, we also conducted a secondary set of analyses to compare groups of participants across the three experimental contexts (methods and results are reported in Online Resource 2, ESM_2).

To compare between-subject effects of ADHD and Autism on resting-state HRV, we carried out separate one-way ANOVAs on CSI and CVI calculated for the 3-min resting-state period, with ADHD and Autism as between-subject factors. Next, we conducted separate mixed ANOVAs on CSI and CVI measured during the passive oddball task to investigate between-subject effects of ADHD and Autism. We also controlled for possible differences between the first and second task blocks by modelling a within-subjects factor Time (2-levels; block 1 and block 2), and we evaluated the presence of any differences between the breaks and the task blocks by modelling a second within-subjects factor Block Type (2-levels; task blocks and breaks). Thirdly, we conducted separate mixed ANOVAs on CSI and CVI for the POP task, with ADHD and Autism included as between-subject factors. We investigated differences between the breaks and the task blocks by modelling the within-subject factor Block Type (2 levels, task blocks vs breaks), and we controlled for any time-related differences between the first and second task half, and between each block of the task, by modelling Task Half (2-levels; 1st and 2nd half of the task) and Block Number (4-levels; blocks 1, 2, 3 and 4) as within-subject factors. In all models, we controlled for the effects of age, gender, verbal IQ and performance IQ.

To replicate previous studies indicating an inverse relationship between CSI and CVI (Bourdon et al., 2018; Oliveira, et al., 2019), we carried out bivariate correlations between the two measures obtained in the three experimental tasks, and we calculated the CVI/CSI ratio to assess the balance between activity in the PNS and in the SNS. Based on our findings showing that ADHD and autism were associated with different autonomic arousal profiles (reported in “Results”), we carried out a two-step cluster analysis on standardised CSI and CVI (averaged across the three experimental contexts) to verify the presence of sub-groups of children with different arousal profiles, among those with ADHD, autism or co-occurring autism and ADHD (we did not include neurotypical children in this analysis). Instead of a-priori deciding the number of clusters to be extracted, the two-step cluster analysis investigates any possible combinations in the data (pre-clustering) before extracting the best combination (the final estimate of clusters) by using the Log-likelihood distance measure and the Bayesian Information Criterion (BIC) criterion.

In order to determine whether the newly created clusters were clinically meaningful, we conducted a multivariate analysis of variance (MANOVA) on measures of ADHD (CRS-3; DAWBA) and autistic symptomatology (SCQ; DAWBA); psychopathology (generalised and social anxiety, oppositional defiant disorder (ODD), and conduct disorder (CD), measured through the DAWBA); and global functioning (CGAS), controlling for gender, verbal and performance IQ, and age, and using the newly created clusters as the between-subject factor. Statistically significant univariate ANOVAs were followed up by analysing pairwise comparisons, for which p-values were adjusted to control for multiple comparisons (using the Benjamini-Hochberg/FDR method described above).

## Results

### Sample Characteristics

106 children and adolescents were included in the final database of participants (mean age = 10.81 years, SD = 2.06 years; 66% males). Among these, 31 children were assigned to the control group of neurotypical (NT) participants; 18 had a diagnosis of autism (but not ADHD), 33 met criteria for co-occurring autism and ADHD (Autism + ADHD), while 24 children had a diagnosis of ADHD without autism. Table 1 summarises the main characteristics of the sample, including the number of participants displaying co-occurring symptoms of anxiety, depression, conduct disorder/oppositional defiant disorder and tics, for each group. Overall, we obtained reliable HR data from 77 children for the resting-state, 85 children for the passive auditory oddball task, and 78 children for the active POP task.

**Table 1:** Main socio-demographic and clinical characteristics of the sample

	Neurotypical (NT)	Autism-only	ADHD-only	Autism + ADHD	Group differences
N	31	18	24	33	–
N Females (%)	13 (41.94%)	7 (38.89%)	8 (33.33%)	8 (24.24%)	–
Age (years) [SD]	10.89 [2.45]	10.91 [2.09]	10.57 [2.25]	10.86 [1.51]	None
WASI – FSIQ [SD]	116.26 [13.09]	104.61 [15.64]	108.12 [11.65]	101.85 [19.02]	Autism + ADHD < NT
SCQ – Total score [SD]	5.10 [7.64]	19.11 [5.98]	15.29 [6.83]	21.06 [6.16]	NT < ADHD, autism, autism + ADHD; Autism + ADHD > ADHD
CRS-3 – ADHD Global Index (T score) [SD]	47.97 [8.36]	79.44 [12.59]	87.96 [4.18]	87.21 [5.26]	NT < autism < ADHD, autism + ADHD
DAWBA CGAS—Global functioning score	79.74 [28.10]	40.11 [11.09]	41.88 [14.40]	38.48 [10.49]	NT > autism, ADHD, autism + ADHD
*Co-occurring diagnoses (N per group)*					
Anxiety	–	10 [56%]	6 [25%]	15 [45%]	
Depression	–	3 [17%]	1 [4%]	6 [18%]	
CD/ODD	–	11 [61%]	17 [71%]	22 [67%]	
Tics	–	1 [6%]	2 [8%]	4 [12%]	

Means [SD] are reported for each group

### Effects of Autism and ADHD on Heart Rate Variability

#### Resting-state (3 min)

Children with ADHD (ADHD-only and autism + ADHD) had significantly lower CSI than those without ADHD (autism-only and neurotypical controls) during the 3-min-long resting-state period (main effect of ADHD: F_1,69_ = 8.687; p = 0.004; η_p_^2^ = 0.112). No other main effects or interactions were statistically significant.

#### Passive Auditory Oddball Task

We found a significant main effect of ADHD on CSI measured during the oddball task (F_1,72_ = 4.786; p = 0.032; η_p_^2^ = 0.062). Children with ADHD (ADHD-only and autism + ADHD) had reduced CSI compared to children without ADHD (NT and autism-only). In addition, there was a significant interaction between Block Type, Autism and ADHD factors (F_1,72_ = 6.281; p = 0.014; η_p_^2^ = 0.080), reflecting that children with ADHD-only had significantly reduced CSI specifically during the 30-s breaks compared to the other groups, including neurotypical controls (p = 0.033; BH-corrected), children with autism-only (p = 0.018; BH-corrected) and children with co-occurring autism and ADHD (p = 0.036; BH-corrected). We also found a significant interaction between Time * Block Type * Autism on CVI during the oddball task (F_1,74_ = 4.235; p = 0.043; η_p_^2^ = 0.054), which showed that children with autism (autism-only and autism + ADHD) had significantly reduced CVI in comparison to children without autism (NT and ADHD-only) during the initial 30-s pre-block break (p = 0.033) and during the second task block (p = 0.038). Across all participants, both CSI and CVI were greater during the blocks of the oddball task, in comparison to the breaks (effect of Block Type; CSI: F_1,72_ = 107.829; p < 0.001; η_p_^2^ = 0.600; CVI: F_1,74_ = 40.721; p < 0.001; η_p_^2^ = 0.355), and CSI was greater in the second block compared to the first (effect of Time: F_1,72_ = 11.719; p = 0.001; η_p_^2^ = 0.140). No other significant main effects or interactions were found.

#### Active POP Task

A significant main effect of Autism was found on CVI during the POP task (F_1,52_ = 4.895; p = 0.031; η_p_^2^ = 0.086), indicating that children with autism (autism-only and autism + ADHD) had reduced CVI during the entire task, compared to children without autism (NT and ADHD-only). Across all participants, CSI was increased during the breaks of the POP task compared to the task blocks (main effect of Block Type: F_1,48_ = 35.834; p < 0.001; η_p_^2^ = 0.427). No other significant main effects or interactions were found.

In addition to the analyses just presented, we analysed between group differences on mean heart rate and HRV across the three experimental tasks in a single statistical model (results are presented in Online Resource 3 and they reflect the results presented above).

### Relationships Between HRV Indices And Clinical Measures

We investigated bivariate correlations between CSI and CVI, autistic and ADHD symptomatology, and global functioning measures, on the sub-sample of participants from the clinical groups (54 children: 15 children with autism-only, 22 children with co-occurring autism and ADHD, and 17 children with ADHD-only). We found negative correlations between all indices of CSI and CVI, even when measured across different experimental conditions (all r < − 0.584; all p < 0.001). We therefore decided to average CSI and CVI indices across the three experimental contexts and ran a correlation analysis between averaged CSI and CVI. Averaged CSI was highly negatively correlated with averaged CVI (r = − 0.676, p < 0.001) indicating that children who had higher values on one of the HRV indices had lower values on the other index. As shown in Table 2 (where we only report statistically significant correlations), more severe hyperactivity and impulsivity (but not inattention) were associated with reduced CSI and increased CVI; conversely, more severe symptoms of autism (especially social-communication deficits) were associated with reduced CVI and increased CSI. Lastly, those children who demonstrated higher global functioning displayed increased CVI. No other correlations were statistically significant.

**Table 2:** Significant bivariate correlations between clinical symptoms, global functioning, CSI and CVI, in children with autism, ADHD and autism + ADHD

	CSI				CVI			
	r	p	Lower 95% C.I	Upper 95% C.I	r	p	Lower 95% C.I	Upper 95% C.I
CRS-3 Hyperactivity Impulsivity	− 0.299*	0.028	− 0.524	− 0.034	0.314*	0.021	0.05	0.536
DAWBA – Autistic symptomatology					− 0.325*	0.016	− 0.545	− 0.063
SCQ Communication	0.364**	0.007	0.104	0.577	− 0.386**	0.004	− 0.594	− 0.129
SCQ Social	0.325*	0.017	0.06	0.547	− 0.487***	< 0.001	− 0.669	− 0.25
SCQ total score	0.329*	0.015	0.068	0.549	− 0.417**	0.002	− 0.616	− 0.168
DAWBA CGAS global functioning score					0.341*	0.013	0.078	0.56

^*^p < 0.05; **p < 0.01; ***p < 0.001

### Clinical Profiles of Children with Different Autonomic Arousal Profiles

The Two-step cluster analysis on averaged CSI and CVI produced two distinct clusters. The majority of clinical participants (39 children) were categorised in Cluster 1, while 15 participants were assigned to Cluster 2. When analysing the characteristics of these clusters, we found that CSI was lower in those assigned to Cluster 1 (mean CSI = 2.453; SD = 0.497) than in children assigned to Cluster 2 (mean CSI = 3.696; SD = 0.752); we therefore labelled Cluster 1 as “Low-CSI”. Moreover, since Cluster 2 had lower mean CVI (mean CVI = 4.205; SD = 0.216) than Cluster 1 (mean CVI = 4.869; SD = 0.230), we labelled Cluster 2 as “Low-CVI”. We also calculated the CVI/CSI ratio, to reflect the balance between activity in the PNS and the SNS, for each child. We found that Cluster 1 (“Low-CSI”) had a higher CVI/CSI ratio (mean = 2.091; SD = 0.585) than Cluster 2 (mean = 1.177; SD = 0.219).

**Fig. 2:**
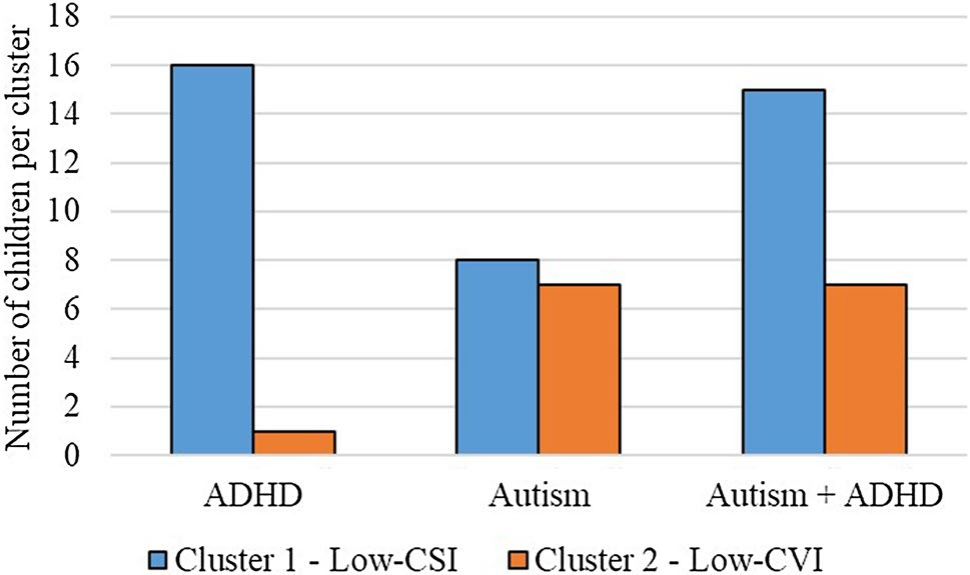
Categorisation of children with autism, ADHD, and autism + ADHD in the clusters ‘Low-CSI’ and ‘Low-CVI’

Almost all children with ADHD-only (16 out of 17) were assigned to Cluster 1, which was characterised by low CSI. Children with autism-only and children with co-occurring autism and ADHD did not show a predominant profile. In fact, some of these children were assigned to the ‘low-CSI’ group (8 out of 15 children with autism-only and 15 out of 22 children with autism + ADHD), while others showed an autonomic arousal profile characterised by low CVI and high CSI (7 out of 15 children with autism-only and 7 out of 22 children with autism + ADHD) (see Fig. 2).

The MANOVA conducted to detect any between-cluster differences on measures of clinical symptomatology was overall significant (F_12,36_ = 3.561; p = 0.002; η_p_^2^ = 0.543) after controlling for age, gender, verbal IQ and performance IQ. Following up this main effect by investigating results of univariate ANOVAs showed significant between-cluster differences in measures of autistic symptomatology, including SCQ Total score (F_1,47_ = 12.334; p = 0.001; η_p_^2^ = 0.208), SCQ Social (F_1,47_ = 12.932; p = 0.001; η_p_^2^ = 0.216), SCQ Communication (F_1,47_ = 11.512; p = 0.001; η_p_^2^ = 0.197) but not SCQ Restricted and Repetitive Behaviours (RRBs) (F_1,47_ = 1.190; p = 0.281; η_p_^2^ = 0.025). We found significant between-cluster differences on CRS Hyperactivity/Impulsivity (F_1,47_ = 5.818; p = 0.020; η_p_^2^ = 0.110), but not on other measures of ADHD symptomatology (CRS global index: F_1,47_ = 0.006; p = 0.939; η_p_^2^ < 0.001; CRS inattention: F_1,47_ = 1.424; p = 0.239; η_p_^2^ = 0.029). There were also significant differences between the two clusters on generalised anxiety (F_1,47_ = 5.381; p = 0.025; η_p_^2^ = 0.103), social anxiety (F_1,47_ = 5.911; p = 0.019; η_p_^2^ = 0.112) and global functioning (CGAS total score: F_1,47_ = 6.102; p = 0.017; η_p_^2^ = 0.115). There was no statistically significant between-cluster difference on ODD (F_1,47_ = 0.563; p = 0.457; η_p_^2^ = 0.012) or CD symptoms (F_1,47_ = 0.020; p = 0.887; η_p_^2^ < 0.001).

As shown in Table 3, after adjusting for multiple comparisons we observed that children with low CVI (Cluster 2) had significantly more severe symptoms of autism (including reduced social abilities and more severe communicative deficits), but reduced hyperactivity/impulsivity compared to those showing low CSI (Cluster 1). Moreover, those with low CVI displayed more severe generalised and social anxiety, and reduced global functioning scores than those with low CSI.

**Table 3:** Significant differences in measures of symptomatology and global functioning between the clusters “Low-CSI” and “Low-CVI”

	Low-CSI	Low-CVI	p-value (adjusted for multiple comparisons; BH/FDR)
CRS–ADHD T-score	84.85	84.63	0.939
CRS–Inattention T-score	82.55	79.23	0.359
CRS–Hyperactivity/Impulsivity T-score	84.62	76.02	0.040*
SCQ Total	15.92	23.42	0.040*
SCQ Social	5.54	9.46	0.040*
SCQ Communication	4.66	7.08	0.040*
SCQ Restricted and Repetitive Behaviours	4.34	5.07	0.375
DAWBA Generalised Anxiety	2.21	2.99	0.043*
DAWBA Social Anxiety	1.70	2.74	0.040*
DAWBA Oppositional Defiant Disorder	3.80	3.46	0.548
DAWBA Conduct Disorders	2.12	2.18	0.939
DAWBA CGAS Global functioning	44.11	37.28	0.040*

^*^Statistically significant between-cluster difference

## Discussion

In the present study, we compared measures of heart rate variability (HRV), the Cardiac Sympathetic Index (CSI) and the Cardiac Vagal Index (CVI) between groups of children with autism, ADHD, co-occurring autism and ADHD, and neurotypical children; in three separate experimental tasks (a resting-state period, a passive attention task and an active response conflict task).

As initially predicted, we found ADHD- and autism-specific effects on HRV, and these were dependent on experimental context. We found evidence of hyper-arousal in children with autism and hypo-arousal in children with ADHD, in line with the main hypotheses of the study. In children with autism, indices of hyper-arousal were found during both passive and active tasks (but not during resting-state) and were associated with reduced activity of the parasympathetic nervous system (i.e., reduced CVI). Conversely, in children with ADHD indices of hypo-arousal (including reduced HR in those with ADHD-only) were found during the resting-state period and the passive auditory oddball task; and were more associated with reduced activity of the sympathetic nervous system (i.e., reduced CSI). Children with co-occurring autism and ADHD did not display a unique profile distinct from autism- and ADHD-only but showed the same atypicalities found in children with ‘pure’ conditions, namely reduced CVI during the POP task and some sections of the passive oddball task (like children with autism-only) and reduced CSI during the initial resting-state period and the oddball task (like children with ADHD-only). These findings, at least in the domain of arousal dysregulation, support the additive model of autism/ADHD comorbidity.

Across the whole sample of participants, we found increased sympathetic and parasympathetic activation during passive auditory stimulation (i.e., the task blocks of the passive auditory oddball paradigm) in comparison to the 30-s periods without sounds (pre-block break). This is partly in line with our hypotheses (we only expected to observe increased sympathetic activation but not parasympathetic activation in relation to task blocks versus breaks). In support of our hypotheses and previous studies that proposed a direct relationship between increased parasympathetic activation and sustained attention during tasks challenging executive function (Minzenberg et al., 2008), we also observed that sympathetic activity was reduced during the blocks of the active POP task, suggesting increased parasympathetic regulation in comparison to the breaks, when children could rest for about one minute before starting the task again.

Guided by the Research Domain Criteria (RDoC) framework (Cuthbert & Insel, 2013), we conducted a cluster analysis and identified two subgroups of children that were differentiated based on their autonomic arousal profile. We found that almost all children with ADHD-only (except one) showed a profile characterised by reduced activity of the SNS, hence hypo-arousal; and this further supports our initial hypotheses of autonomic hypo-arousal in ADHD and is consistent with previous research (Bellato et al., 2020). Conversely, among children with autism (with or without ADHD) heterogeneous arousal profiles were found, with some children displaying hypo-arousal (low CSI and high CVI) and others displaying hyper-arousal (high CSI and low CVI). This is again in line with previous literature that proposed the presence of subgroups of autistic children with opposite autonomic arousal profiles, i.e., hyper- or hypo-arousal (Arora et al., 2021; Hirstein et al., 2001; Mathersul et al., 2013; Schoen et al., 2008).

Our findings are consistent with the published literature and previous studies, demonstrating that ADHD (especially when it presents without co-occurring autism) is likely to be characterised by reduced functioning of the SNS and hypo-arousal, especially during less stimulating situations (Bellato et al., 2020). In such situations, hyperactivity and impulsivity might act as homeostatic compensatory mechanisms to upregulate arousal (Geissler et al., 2014). While this prediction still needs to be tested and confirmed in empirical studies, we found some indirect support for this idea in our data as reduced CSI (reflecting reduced activity of the SNS) was associated with higher presence of hyperactive/impulsive symptoms among children with ADHD and autism. Our findings of reduced parasympathetic activation and hyper-arousal in at least a subgroup of autistic individuals (with or without ADHD) are in line with previous studies (Lydon et al., 2016; Porges et al., 2013). Hyper-arousal in autism might be associated with excessive responsiveness to sensory stimulation, and autistic individuals with this profile might find it difficult to down-regulate autonomic arousal in specific environments. However, this putative association in autism needs further investigation. Therefore, it would be interesting to investigate whether condition-specific symptoms like Restricted/Repetitive Behaviours or sensory/social avoidance in autism and hyperactivity/impulsivity in ADHD are strategies implemented by people with these conditions when they need to down- or up-regulate a dysregulated state of arousal. For example, it should be tested if implementing such proposed compensatory homeostatic mechanisms is associated with short-term changes on objective measures of autonomic arousal, including HRV, HR, or electrodermal activity.

There are some limitations of the study which should be addressed in future research. Firstly, this was an experimental study based in a laboratory and so the findings might not fully generalise to more naturalistic environments in the children’s daily lives such as the classroom or home environments. In addition, it is important to acknowledge that the laboratory environment may have influenced HRV, particularly as ADHD and autism are both associated with increased rates of anxiety. Although the design of the study was appropriate to address our research questions, it will be important in future research to measure autonomic arousal in children with ADHD and/or autism in naturalistic settings, for example using wearable sensors or other digital devices. This would also enable data collection in much larger samples than those reported here, improving the generalisability of findings on autonomic arousal in these populations.

Secondly, although we were able to test 106 children and adolescents by adopting a broad recruitment strategy aimed at recruiting an heterogenous sample (for example, we involved parents from support groups, teachers, and SENCOs in special education schools), not every child completed all tasks. Specifically, several children completed the resting-state and the passive auditory oddball task but decided to not proceed with the active task because they were too tired or did not want to continue. This could indicate that arousal regulation was particularly challenging for these children and so their attrition from the study before the final task may have influenced the findings. Moreover, although we designed our study to include children with low intellectual or adaptive functioning or more severe clinical profiles (for example by splitting the sessions or allowing more time for the child to familiarise with the lab setting in advance to the visit), parents of these children reported that they did not consider the study suitable for their child. Therefore, our findings may not be generalizable to children with co-occurring intellectual disability, sensory difficulties, high levels of anxiety or reduced global functioning. Further research is needed to understand more fully the barriers faced by these children in participating in neuroscientific research so that experiments can be adapted to be more inclusive or can be conducted in settings where children feel more able to participate.

Lastly, participants with ADHD on stimulant medication were required to withdraw their medication at least 24 h prior to the testing session (to avoid any confounding effect of medication on HRV). This meant that we could not include children on non-stimulant ADHD medication (e.g., noradrenergic agonists) because non-stimulants require a longer washout period and cannot be withdrawn easily. As a result, the findings may not be generalisable to children with ADHD (with or without ASD) who are prescribed non-stimulant medication possibly because they tolerate stimulants less well. Considering that autonomic side effects may be one reason why children with ADHD tolerate one class of medication better than another, differences between medications and their effects on arousal and cognition remains an important empirical question which should be addressed in future research.

It would be also important to further investigate the associations between core domains of human functioning (within the RDoC research framework; Cuthbert & Insel, 2013) and transdiagnostic domains of clinical symptoms which impact adaptive or global functioning (including but not limited to anxiety and other mood dysregulations, disruptive and hyperactive behaviours). The present study in fact demonstrated that, irrespectively of having a diagnosis of autism and/or ADHD, children who were towards the hypo-end of the autonomic arousal continuum showed more hyperactive-impulsive behaviours (supporting the hypothesis of hyperactivity and impulsivity as compensatory homeostatic mechanisms to up-regulate hypo-arousal) but better global functioning than those on the hyper-end of the continuum, who displayed more severe socio-emotional difficulties and autistic symptoms, and higher anxiety.

Focusing on RDoC domains (such as arousal and arousal regulation) and subgrouping individuals with different profiles based on such domains, might better explain heterogeneity in symptomatology, adaptive or global functioning, and interventional outcomes (Cuthbert & Insel, 2013). Stratifying individuals into subgroups with similar autonomic arousal profiles (irrespective of their diagnoses) might also be useful to aid more precise characterisation of the difficulties of individual children, and to facilitate the targeting of pharmacological and behavioural interventions on these difficulties, rather than the current approach to intervention which is primarily led by the diagnostic label assigned to the child. Furthermore, improving arousal regulation in these children, especially when autism and ADHD co-occur in the same individual or the behavioural/symptomatologic phenotype is complex, might have positive effects on both the clinical symptomatology and on adaptive and global functioning.

## Conclusions

In the present study, we found evidence of hyper-arousal in autism, reflecting reduced activity of the parasympathetic nervous system especially during more demanding tasks and activities, and hypo-arousal in ADHD, associated with reduced functioning of the sympathetic nervous system especially during resting-state and less engaging tasks. We also found that children with a diagnosis of autism or ADHD could be sub-grouped based on their autonomic arousal profile; and that those with a profile of hyper-arousal displayed more severe social and communication difficulties, increased anxiety, and reduced global functioning. Based on these findings, we suggest that future research should focus on further clarifying the relationship between dysregulated autonomic arousal and transdiagnostic symptomatologic domains (including anxiety, behavioural difficulties, mood and emotional dysregulation), which might affect adaptive, global and cognitive functioning and treatment response in autism and ADHD.

## Supplementary Information

Below is the link to the electronic supplementary material.

Supplementary file1 (DOCX 88 KB)

Supplementary file2 (DOCX 16 KB)
