# Supplementary material for: Heart Rate Variability in Children and Adolescents with Autism, ADHD and Co-occurring Autism and ADHD, During Passive and Active Experimental Conditions
Source: J Autism Dev Disord. 2021 Oct 30;52(11):4679–91. doi: 10.1007/s10803-021-05244-w (PMC9556357; doi:10.1007/s10803-021-05244-w)
Supplement: Supplementary file 1 — Supplementary file1 (DOCX 88 KB) [file 10803_2021_5244_MOESM1_ESM.docx]

# ESM_1 - Procedure and mathematical formulas for calculation of CSI and CVI

In order to calculate CSI and CVI, a Poincaré plot is created by plotting every peak-to-peak interval against the preceding interval, which results in a two-dimensional graphical ellipsoid-shaped cloud of points, as represented in Figure S1. Two main parameters of this ellipsoid graph, i.e., SD1 and SD2, can be mathematically extracted from the distribution of R-R-intervals in a specific time window. Considering the line of identity as the 45° oriented line representing the identity I_k_ = I_k+1_, SD1 is a measure of the dispersion of the points perpendicularly to the line of identity (i.e., the width of the ellipse), while SD2 represents the dispersion of points along the identity line (i.e., the length of the ellipse) (see Figure S1). More specifically, the mathematical calculations of SD1 and SD2 were carried out using the following equations:

$$\text{SD1}=SD(\frac{1}{\sqrt{2}}\text{I}\text{(k)}-\frac{1}{\sqrt{2}}\text{I}\text{(k+1}\text{)})$$

$$\text{SD2}=SD(\frac{1}{\sqrt{2}}\text{I}\text{(k)}+\frac{1}{\sqrt{2}}\text{I}\text{(k+1)})$$

with *k* = 1, 2, 3, …, (*n* - 1); and *n* = number of cardiac beats within the period.

SD = standard deviation of the sample

By multiplying the SD1 and the SD2 by four, it is possible to obtain an estimation of the transverse length (T) and the longitudinal length (L) of the ellipse, which are further used to calculate the CSI and the CVI (Toichi et al., 1997), as following:

$$\text{CSI}=\frac{L}{T}$$

$$\text{CVI}=\log_{10} (L\times T)$$

with *T* = 4 x SD1 and *L* = 4 x SD2


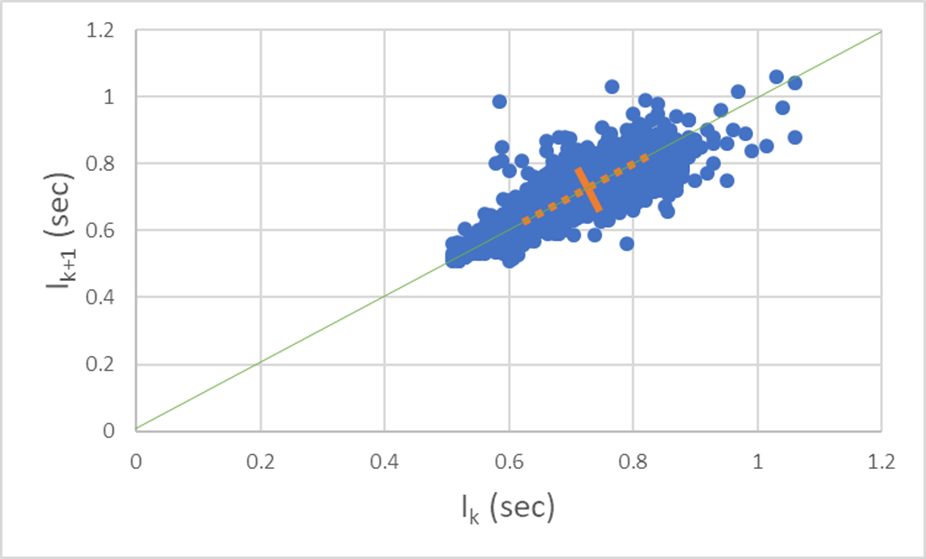


Figure S1. Example representation, based on collected HR data, of a Poincaré plot.

Green line: identity line. Straight orange line: SD1; Dotted orange line: SD2.
