# Supplementary material for: Heart Rate Variability in Children and Adolescents with Autism, ADHD and Co-occurring Autism and ADHD, During Passive and Active Experimental Conditions
Source: J Autism Dev Disord. 2021 Oct 30;52(11):4679–91. doi: 10.1007/s10803-021-05244-w (PMC9556357; doi:10.1007/s10803-021-05244-w)
Supplement: Supplementary file 2 — Supplementary file2 (DOCX 16 KB) [file 10803_2021_5244_MOESM2_ESM.docx]

# ESM_2 - Differences in Heart Rate and HRV across the three tasks

## Heart Rate

In addition to the analyses presented in the manuscript, we analysed mean HR to give further insight into general functioning of the autonomic system in the sample: reduced HR is interpreted as an index of hypo-arousal, while increased HR reflects hyper-arousal. A repeated measures ANOVA on average HR was carried out with Context (3-levels: resting-state, ‘active’ and ‘passive’ task) as the within-subjects factor. ADHD and Autism were added to the models as between-subjects factors (each with two levels: present, absent) and we controlled for the effects of age, gender, verbal and performance IQ.

We found significant between-subject effects of Autism (F_1,58_ = 5.113; p = 0.028; η_p_^2^ = 0.081) and ADHD (F_1,58_ = 4.905; p = 0.031; η_p_^2^ = 0.078) on average heart rate during the three experimental tasks, but no significant main effect of Task (F_1.446,83.848_ = 2.282; p = 0.123; η_p_^2^ = 0.038). We carried out between-groups pairwise comparisons and found that children with ADHD-only had overall lower average HR compared to neurotypical children (p = 0.034; BH-corrected), children with autism-only (p = 0.018; BH-corrected) and children with autism + ADHD (p = 0.034; BH-corrected) (see Table S1).

## Heart Rate Variability

We carried out separate mixed ANOVA on CSI and CVI with Context (resting-state; ‘passive’ task; ‘active’ task) as the within-subject factor. ADHD and Autism were added to the models as between-subjects factors (each with two levels: present, absent) and we controlled for the effects of age, gender, verbal and performance IQ.

When investigating CSI and CVI across the tasks, we did not find any effect of Context on CSI (F_1.330,87.793_ = 0.765; p = 0.467; η_p_^2^ = 0.011) or CVI (F_1.407,92.864_ = 1.261; p = 0.279; η_p_^2^ = 0.019), but we found a main effect of ADHD on CSI (F_1,66_ = 5.791; p = 0.019; η_p_^2^ = 0.081). Specifically, across the three tasks children with ADHD showed lower CSI (M = 2.718; S.E. = 0.118) compared to those without ADHD (M = 2.298; S.E. = 0.118). However, we also find a marginally significant interaction between ADHD and Context (F_1.330,87.793_ = 3.489; p = 0.053; η_p_^2^ = 0.050) showing that, in line with the result already presented, children with ADHD displayed lower CSI that those without ADHD during resting-state (Mean difference = 0.675; p = 0.004) and the passive task (Mean difference = 0.375; p = 0.049) but not during the active task (Mean difference = 0.209; p = 0.275). No other significant main effects or interactions were found.

Table S1. Mean heart rate (in BPM) during the testing session (for each experimental group)

| Group | Mean heart rate | Standard Error | 95% Confidence Interval | |
| --- | --- | --- | --- | --- |
|  |  |  | Lower Bound | Upper Bound |
| NT | 87.706 | 2.580 | 82.543 | 92.870 |
| ADHD-only | 78.014 | 2.837 | 72.335 | 83.693 |
| Autism-only | 90.238 | 2.786 | 84.661 | 95.815 |
| Autism + ADHD | 87.644 | 2.493 | 82.654 | 92.635 |
